# Hepatic leptin receptor expression can partially compensate for IL-6Rα deficiency in DEN-induced hepatocellular carcinoma

**DOI:** 10.1016/j.molmet.2018.08.010

**Published:** 2018-09-05

**Authors:** Melanie J. Mittenbühler, Hans-Georg Sprenger, Sabine Gruber, Claudia M. Wunderlich, Lara Kern, Jens C. Brüning, F. Thomas Wunderlich

**Affiliations:** 1 Max Planck Institute for Metabolism Research, Center for Endocrinology, Diabetes and Preventive Medicine (CEDP), Cologne, 50931, Germany; 2 Excellence Cluster on Cellular Stress Responses in Aging Associated Diseases (CECAD), Germany; 3 Max Planck Institute for Biology of Ageing, Cologne, 50931, Germany

**Keywords:** Hepatic leptin receptor, Ob-R, Hepatocellular carcinoma, IL-6Rα deficiency, Obesity

## Abstract

**Objective:**

The current obesity pandemic represents a major health burden, given that it predisposes to the development of numerous obesity-associated disorders. The obesity-derived adipokines not only impair systemic insulin action but also increase the incidence of hepatocellular carcinoma (HCC), a highly prevalent cancer with poor prognosis. Thus, worldwide incidences of HCC are expected to further increase, and defining the molecular as well as cellular mechanisms will allow for establishing new potential treatment options. The adipose tissue of obese individuals increases circulating leptin and interleukin-6 (IL-6) levels, which both share similar signaling capacities such as Signal Transducer and Activator of Transcription 3 (STAT3) and Phosphoinositide 3-kinase (PI3K)/Akt activation. While mouse models with deficient IL-6 signaling show an ameliorated but not absent Diethylnitrosamine (DEN)-induced HCC development, the morbid obesity in mice with mutant leptin signaling complicates the dissection of hepatic leptin receptor (LEPR) and IL-6 signaling in HCC development. Here we have investigated the function of compensating hepatic LEPR expression in HCC development of IL-6Rα-deficient mice.

**Methods:**

We generated and characterized a mouse model of hepatic LEPR deficiency that was intercrossed with IL-6Rα-deficient mice. Cohorts of single and double knockout mice were subjected to the DEN-HCC model to ascertain liver cancer development and characterize metabolic alterations.

**Results:**

We demonstrate that both high-fat diet (HFD)-induced obesity and IL-6Rα deficiency induce hepatic *Lepr* expression. Consistently, double knockout mice show a further reduction in tumor burden in DEN-induced HCC when compared to control and single LepR^L−KO^/IL-6Rα knock out mice, whereas metabolism remained largely unaltered between the genotypes.

**Conclusions:**

Our findings reveal a compensatory role for hepatic LEPR in HCC development of IL-6Rα-deficient mice and suggest hepatocyte-specific leptin signaling as promoter of HCC under obese conditions.

## Highlights

• High fat diet feeding induces LEPR expression in hepatocytes.
• IL-6Rα deficiency induces LEPR expression in hepatocytes.
• Hepatic LEPR deficiency fails to affect body composition and metabolism.
• Hepatic LEPR deficiency ameliorates HCC burden in IL-6Rα-deficient mice.

## Graphical abstract

**Figure:**
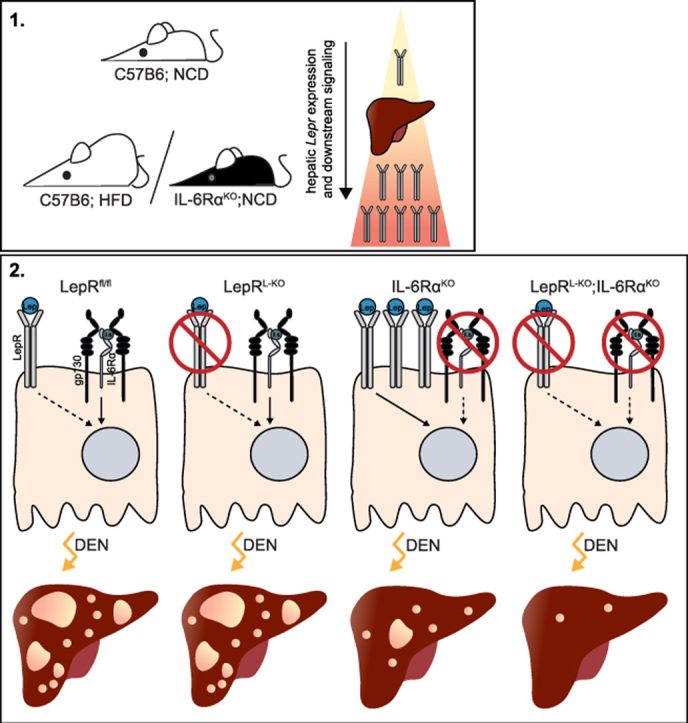

## 1 Introduction

Hepatocellular carcinoma (HCC) is one of the most prevalent causes of cancer deaths, owing to limited available therapeutic strategies [1], [2]. HCC is defined as inflammation-driven and obesity-associated cancer with increased incidences over the last decades that might be in part a consequence of the steadily increasing obesity epidemic in the westernized world [3], [4], [5], [6], [7], [8]. Despite extensive ongoing research on obesity-associated cancer development, numerous contributing factors still remain elusive.

Besides local variation of HCC incidences, gender-specific manifestations of HCC were observed with higher rates in males compared to females [9]. This fact might be a consequence of higher hepatitis B and hepatitis C virus infection rates and increased alcohol consumption of male individuals [4], [10]. However, studies in mice further revealed the sexual dimorphism in HCC after chemical induction via low estrogen levels in males, which can directly interfere with immune response mechanisms [11], [12]. In line with this evidence, studies in mice have shown that the degree of inflammation strongly correlates with HCC burden [11], [13], [14].

Obesity was identified as a state of chronic, low-grade inflammation due to systemic elevated expression levels of tumor necrosis factor alpha (TNFα) and IL-6 [15], [16], [17], [18], [19], [20]. Hence, the relative risk of dying from cancers is increased upon obesity and, amongst all cancer types, liver cancer displays the highest mortality risk of males with increased body mass index (BMI) in the U.S. [5]. Mouse models of HCC recapitulate this increase in tumor burden upon genetically and dietary-induced obesity as a consequence of obesity-induced inflammation [22]. However, while IL-6 deficiency ameliorates DEN-induced HCC in lean and obese mice, inactivation of IL-6 receptor alpha (IL-6Rα) reduces DEN-induced tumor burden only in lean mice, suggesting a compensatory overlapping signaling cascade in obese IL-6Rα-deficient mice [11], [23]. Notably however, lean IL-6- and IL-6Rα-deficient mice still develop fewer HCCs [11], [23].

On the one hand, IL-6 binds to its membrane-bound receptor composed of IL-6Rα and gp130 to initiate classical IL-6 signaling. On the other, a trans-signaling mechanism exists where IL-6Rα is shedded from the cell surface to create a soluble IL-6Rα form that renders cell types that do not express IL-6Rα to be IL-6-responsive [24], [25], [26], [27]. Both signaling cascades are essential for liver regeneration and hepatocyte proliferation mainly via their downstream action on STAT3, which was shown to be over-activated in inflammation-associated cancerogenesis [28]. In fact, blockade of IL-6 trans signaling by the designer cytokine sGP130Fc prevents tumor proliferation and angiogenesis in HCC at least in part via its inhibitory action on Stat3 [29]. Consistently, interfering with hepatic STAT3 expression impairs hepatocarcinogenesis and hepatic regeneration [30], [31], [32]. Besides Stat3 downstream signaling, IL-6 also activates Phosphoinositide 3-kinase (PI3K)/Akt pathway, which was shown to promote cancer progression [33].

Apart from IL-6, other cytokines, growth factors, and hormones also utilize the same downstream signaling pathways as IL-6 and could compensate for IL-6Rα deficiency [34]. Such a compensating factor could be leptin, which is expressed by adipocytes proportionally to body fat mass [35]. Leptin binding to its class 1 cytokine receptor (LEPR) triggers the activation of intracellular JAK/STAT3 signaling as well as PI3K/Akt downstream signaling [36], [37], [38], [39], [40], [41]. Six different isoforms of the LEPR were identified, (LEPRa-f); however, only the long form, LEPRb, is capable of intracellular signal transduction [37], [42], [43]. Leptin plays a major role in the regulation of energy homeostasis and has been strongly associated with obesity [44]. Leptin signaling regulates energy homeostasis predominantly via its action on neurons of the hypothalamus, where it triggers the release of anorexigenic peptides to regulate hunger and satiety [45], [46]. Consistently, mice lacking leptin (*ob*/*ob* mice) or the LEPR (*db*/*db* mice) are characterized by hyperphagia and decreased energy expenditure, resulting in severe morbid obesity [47], [48], [49].

However, the LEPR is not only expressed on hypothalamic neurons, but also in peripheral tissues, for instance the liver [50]. Interestingly, leptin signaling is linked to tumor development in various types of tissues [51], [52]. Upon leptin stimulation, hepatic LEPR expression is increased to generate a soluble form of the LEPR that can dampen the amount of circulating leptin [50]. In the liver, leptin and its receptor are pro-inflammatory and pro-fibrogenic, thereby potentially affecting HCC progression [53], [54], [55]. However, despite the potential oncogenic function of leptin via its capacity to regulate JAK/Stat3 signaling, the role of leptin in HCC has not been investigated yet.

Here we aim to ascertain the contribution of compensating hepatic LEPR signaling on chemical-induced HCC development in IL-6Rα-deficient mice. Therefore, we subjected hepatic LEPR (LepR^L−KO^), IL-6Rα whole body (IL-6Rα^KO^), and double-deficient (D-KO) animals to the diethylnitrosamine (DEN)-HCC model. DEN is a genotoxic hepatocarcinogen that causes extensive DNA damage, hepatocyte cell death and compensatory hyperproliferation ultimately resulting in HCC development [56]. Our findings demonstrate that additional ablation of hepatic LEPR further ameliorates HCC burden in IL-6Rα-deficient mice.

## 2 Materials and methods

### 2.1 Animal care

The mice were housed at 22–24 °C in a virus-free animal facility and were exposed to a 12 h light/12 h dark cycle. The animals were fed *ad libitum* normal chow diet (NCD) (Altromin, 1324) or HFD (Altromin, 1057). The access to water was unlimited. At 8 months of age, animals were sacrificed using CO_2_. The experiments were authorized by the local government authorities (case number 84–02.04.2014.A074) and were in accordance with NIH guidelines.

### 2.2 Generation of LepR^L−KO^, IL-6Rα^KO^ mice

The conditional IL-6Rα mouse strain, in which loxP sites flank exons 2 and 3 of the IL-6Rα, has been described previously [57]. Whole body IL-6Rα^KO^ mice have been generated by crossing the loxP-flanked IL-6Rα allele to deleter Cre that was subsequently crossed out in the next breeding step [58]. IL-6Rα^KO^ mice without deleter Cre were crossed to LepR^fl/fl^ animals, generated by McMinn and colleagues [59]. To generate hepatocyte-specific LepR^L−KO^ and D-KO mice, LepR^fl/fl^ or IL-6Rα^KO^, LepR^fl/fl^ animals were crossed to Alfp Cre^tg/wt^, LepR^fl/fl^ animals, respectively. D-KO mice exhibit IL-6Rα deficiency in the whole body and LepR deficiency only in hepatocytes. Hepatocyte-specific LepR inactivation was used here instead of whole body deletion to prevent the recapitulation of the morbid obese phenotype of ob/ob mice; mice were on a mixed C57/BL6NX129 background.

### 2.3 Glucose tolerance test

Fasted mice received an *intraperitoneal* (*i*.*p*.) injection of 20% glucose (10 ml/kg BW, Bela-Pharm). Blood glucose levels were measured 15, 30, 60, and 120 min after glucose injection using a Contour® blood glucose meter (Bayer).

### 2.4 Insulin tolerance test

To evaluate the insulin sensitivity of the animals, an insulin tolerance test (ITT) was performed. Therefore, random fed mice were injected *i*.*p*. with insulin (0.75 U insulin/g BW, Sanofi) and blood glucose was measured using Contour® blood glucose meter (Bayer) 15, 30, and 60 min after injection.

### 2.5 DEN-induced HCC

To induce HCC, male mice were injected *i*.*p*. with 25 mg/kg BW diethylnitrosamine (DEN) (Sigma) at postnatal day P15. Upon weaning, mice were separated into groups of 3–5 animals per cage and housed until the end of the study at 8 months of age. The body weight was controlled weekly.

### 2.6 Organ preparation

After 8 months, mice were sacrificed using a CO_2_ chamber. Heart blood was taken and the liver, WAT, skeletal muscle and the hypothalamus were dissected and snap frozen in liquid nitrogen. Liver tissue was embedded in tissue freezing medium (Jung) for cryo-sections. WAT and liver weight were determined, and liver tumor numbers were counted macroscopically. The organs were stored at −80 °C for further analysis.

### 2.7 ELISA

Serum insulin (mouse Insulin ELISA, Crystal Chem Inc., 90080) and leptin (mouse Leptin ELISA, Crystal Chem Inc., 90030) were determined by ELISA according to manufacturer's instructions.

AST and ALT activity in the serum, as well as Triglycerides and Cholesterol were determined at the diagnostics laboratory/Institute of Clinical Chemistry of the University Hospital Cologne using standard techniques.

### 2.8 Western blot analysis

Liver tissue was homogenized with a bead homogenizer (MP Biomedicals) and tissue as well as cells were lysed in RIPA buffer. Protein concentrations were determined by Pierce BCA protein assay kit (Thermo), Proteins were separated by SDS–polyacrylamide gel electrophoresis (6–10%) and transferred to Nitrocellulose or PVDF membranes (Bio-Rad) using standard protocols. Membranes were probed with the following antibodies: anti-phospho-Stat3 1:500-1:1000 (Tyr705) (#9145, Cell Signaling Technology Inc.), anti-Stat3 1:1000 (#9139, Cell Signaling Technology Inc.), anti-β-Actin 1:5000 (A2228, Sigma–Aldrich), anti-phospho-eIF4E 1:500 (#9741, Cell Signaling Technology Inc.), anti-eIF4E 1:1000 (#9742, Cell Signaling Technology Inc.). Anti-rabbit horseradish peroxidase (HRP) (A6154, Sigma) or anti-mouse HRP (A4416, Sigma) secondary antibodies were used. ImageJ software was used for western blot quantification.

### 2.9 Quantitative PCR

Frozen tissues or cells were homogenized in QIAzol (Qiagen) and RNA was isolated using the RNeasy mini kit (Qiagen) and treated with DNase (79254, Qiagen). RNA was reversely transcribed with High Capacity cDNA Reverse Transcription Kit and amplified by using TaqMan Gene Expression Master Mix (both Applied Biosystems). Relative expression of mRNAs was determined by using standard curves based on cDNA derived from the respective tissues, and samples were adjusted for total RNA content by *TATA-binding protein* (*Tbp*) quantitative PCR. Calculations were performed by a comparative cycle threshold (Ct) method: starting copy number of test samples was determined in comparison with the known copy number of the calibrator sample (ddCt). The relative gene copy number was calculated as 2-ddCt. Quantitative PCR was performed on an ABI Quantstudio Detector (Applied Biosystems). The following TaqMan probes (Applied Biosystems) were used for gene expression assays:

*Il-6rα* (Mm00439653_ml), *Lepr* (Mm01262069_m1), *Lepr* (Mm01265583_m1), *Timp1* (Mm00441818_m1), *Socs3* (Mm00545913_s1), *Srebp1* (Mm00550338_m1), *Srebp2* (Mm01306292_m1), *Dgat1* (Mm00515643_m1), *Dgat2* (Mm00499536_m1), *Scd1* (Mm00772290_m1), *Pparg* (Mm00440945_m1), *Fasn* (Mm00662319_m1), *Gck* (Mm00439129_m1), *Pepck* (Mm00440636_m1), *G6pc* (Mm00839363_m1), *Vegf* (Mm00437304_m1), *Egf* (Mm01316968_m1), *Egfr* (Mm00433023_m1), *Glut1* (Mm00441473_m1), *Myc* (Mm00487804_m1), *Bcl2* (Mm00477631_m1), *Mcl1* (Mm01257352_g1), *Ccl2* (Mm00441242_m1), *Ccl7* (Mm00443113_m1), *Mmp9* (Mm00442991_m1), *Stat3* (Mm00456961_m1), *Tbp* (Mm00446973_m1).

### 2.10 Caspase3 activity assay

Caspase 3 activity was determined by measuring cleaved caspase 3 amounts of liver lysates by using PathScan Cleaved Caspase 3 ELISA kit (Cell Signaling) according to manufacturer's instructions.

### 2.11 Primary murine hepatocyte culture

Mice were perfused 5 min with EBSS solution (4155-048, GIBCO) containing 0.5 mM EGTA via vena cava. Subsequently, mice were perfused with 50 ml 40 °C EBSS (24010-043, GIBCO) containing 10 mM HEPES, 15 mg Collagenase Type II (LS004189, Worthington) and 2 mg Trypsin inhibitor (T9128, Sigma). After perfusion, the liver was transferred to 10 ml ice cold EBSS containing 10 mM HEPES. Hepatocytes were harvested in 10 ml ice cold EBSS by using a cell scraper and were then filtered through a 100 μm nylon strainer and centrifuged for 5 min at 500× *g* at 4 °C. The pellet was washed two times in DMEM High Glucose GlutaMAX (61965-059, GIBCO) containing 1% sodium pyruvate, 1% non-essential amino acids, 5% fetal Calf Serum (FCS), 1% Penicillin/Streptomycin. Subsequently, the pellet was dissolved in 10 ml medium. The viability was determined by trypan blue dye exclusion. 3 × 10^6^ hepatocytes isolated from respective animals, were plated in each well of a 6 well plate (Corning™ BioCoat™) and incubated with medium overnight. For leptin stimulation, cells were incubated for 15, 30, or 60 min with fasting medium containing 1 μg/ml leptin (L3772, Sigma). For IL-6 stimulation, cells were fasted for 4 h and then stimulated with 50 ng/ml IL-6 for 15, 30, and 60 min. Subsequently, whole cell lysates were isolated and used for western blot analysis.

### 2.12 Immunohistochemistry and histological analysis

Liver tissue was embedded in tissue freezing medium (Jung) for frozen block preparation.

To detect proliferating cells, tissue sections were stained with Ki67 antibody (#ab15580, Abcam) and donkey anti-rabbit red (#711-025-152, Jackson). Ki67-positive cells were counted and normalized to DAPI (Biozol) positive nuclei.

To detect pSTAT3 positive cells, tissue sections were stained with pSTAT3 antibody (#9145, Cell Signaling) and goat anti-rabbit HRP (#NEF812001EA, Perkin Elmer). pSTAT3-positive cells were counted and normalized to DAPI (Biozol) positive nuclei.

Lectin staining was performed at room temperature. Liver sections were fixed in 4% PFA and washed 2 times 5 min in PBS. Fluorescein Esculentum (Vector, #FL-1171) was applied for 60 min (1:200 in Signal Stain, Cell Signaling) and subsequently sections were washed 3 times 10 min in PBS containing 0.1% TritonX. Sections were mounted in mounting medium with DAPI (Vectorshield).

Oil-Red-O (Sigma Aldrich, O0625-25G) staining was performed at room temperature for 15 min. Afterwards H&E staining was performed as described below.

H&E (Mayer's Haematoxylin/Erythrosin) staining was performed at room temperature. Sections were incubated in Haematoxylin for 6 min and afterwards washed once short, followed by a 15 min wash in tap water. After a short wash in distilled water, Eosin staining was performed (1 min). The sections were washed 7 times in tap water. Before mounting with Entellan, liver tissue was dehydrated in ascending ethanol concentrations followed by transfer into xylol.

### 2.13 Fluorescent *in situ* hybridization *Lepr* and subsequent antibody F4/80 staining

Frozen livers of NCD and HFD fed animals were cut into 15 μm thick sections on a cryostat. Fluorescent *in situ* hybridization for the detection of *Lepr* mRNA was performed using RNAscope (ACD; Advanced Cell Diagnostics Inc., Hayward, CA). A probe detecting the *Lepr* was utilized (#402731, ACD). To ensure tissue RNA integrity and optimal assay performance, negative and positive controls were processed in parallel with the *Lepr* probe. The sections were pretreated as suggested from ACD (Fresh frozen sample preparation and pretreatment) with the following change: Protease plus was used for digestion for 10 min at 40 °C. RNAscope was performed according to the online protocol for RNAscope Multifluorescent Assay. Subsequently, F4/80 staining was performed. Sections were blocked in 5% goat serum in PBS for 1 h at room temperature. Afterwards they were incubated over night at 4 °C with a rat-anti-F4/80 antibody (1:100, #MCA497G, Serotec). On the following day, the sections were washed 3 times 10 min with PBS and incubated for 1 h at room temperature with an Alexa-594 goat anti-rat-antibody (1:500, #A11007, Invitrogen). After 3 times 10 min wash with PBS, sections were mounted in mounting medium with DAPI (Vectorshield). Images were acquired using a confocal Leica TCS SP-8-X microscope.

### 2.14 Statistical analysis

**Figure 1:**
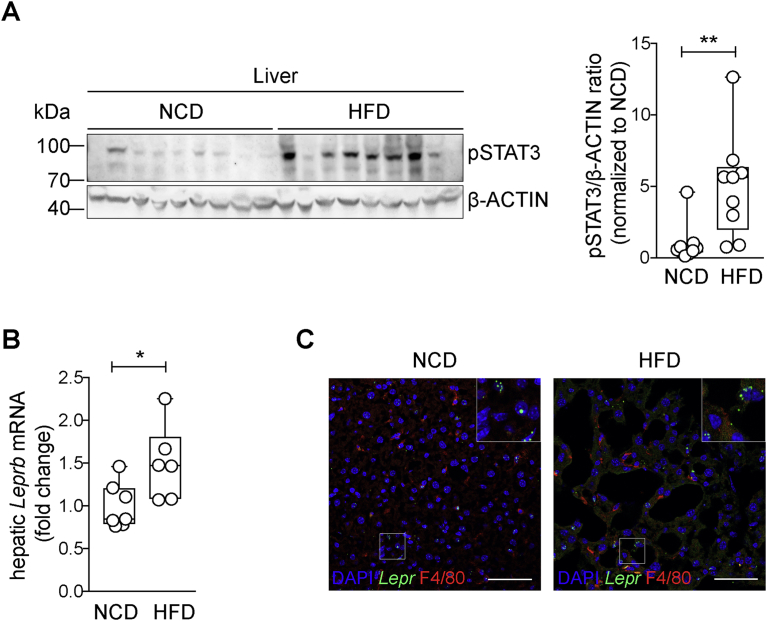
**Hepatic *Lepr* expression is induced upon HFD feeding**. (A) Western blot analysis of basal pSTAT3 level in whole liver lysates isolated from 7-month-old NCD- and HFD-fed mice (n = 9) and respective quantification (two-tailed unpaired t-test). pSTAT3/β-ACTIN ratio normalized to NCD expression. (B) qPCR analysis of *Leprb* mRNA expression in whole liver tissue isolated from 7-months-old NCD- or HFD-fed mice (probe spanning exon 17–18 of long *Leprb*). *Tbp* was used as internal control, normalized to NCD (n = 6–7, two-tailed unpaired t-test). (C) Representative liver sections of NCD- and HFD-fed mice stained with ISH for *Lepr* mRNA and IHC for F4/80. Blue = DAPI, green = *Lepr*, red = F4/80. Scale bar = 50 μm. NCD = normal chow diet, HFD = high-fat diet. Data are means ± Min. and Max. p* ≤ *0*.*05*, p** ≤ *0*.*01*.

**Figure 2:**
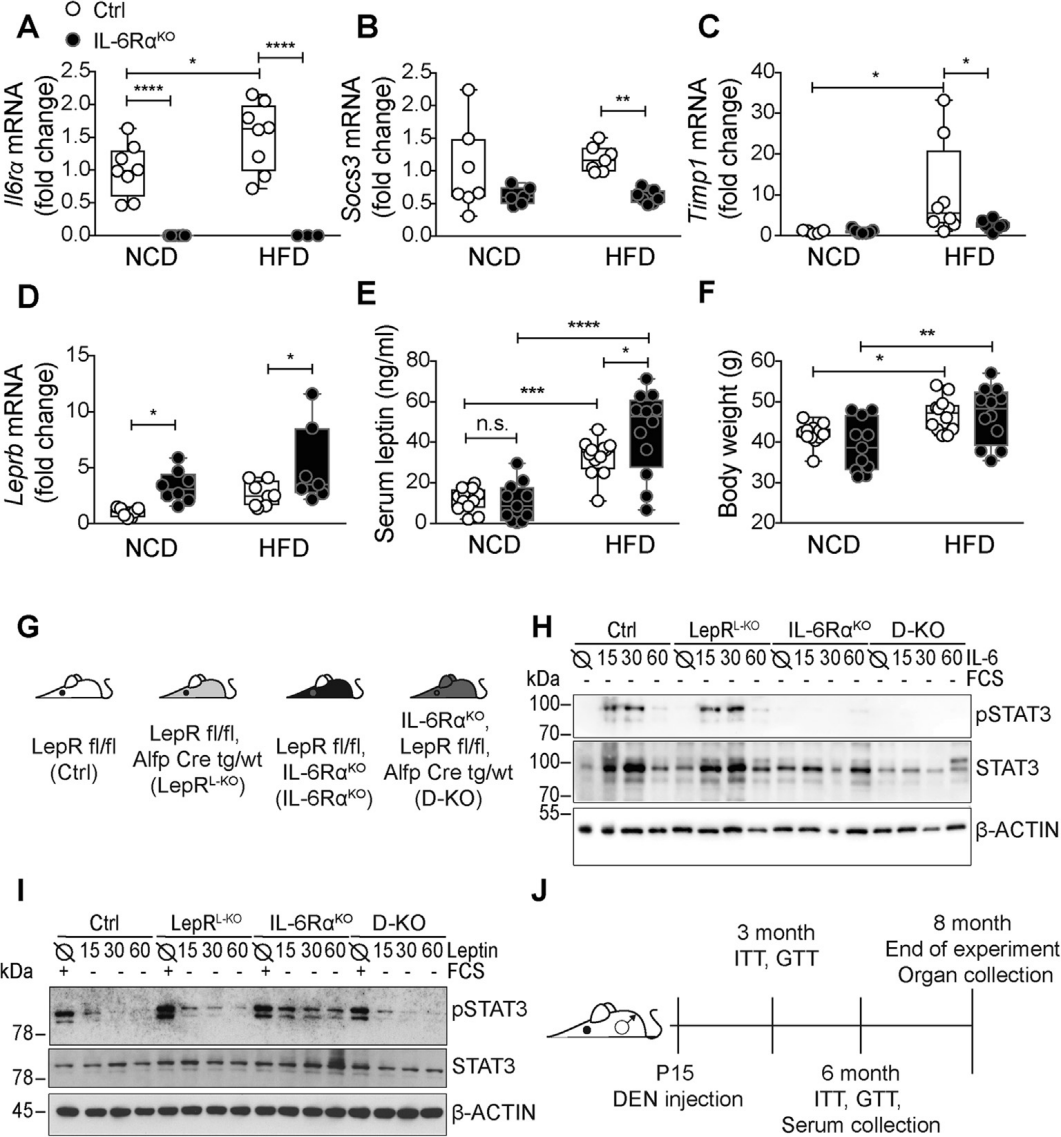
**IL-6Rα deficiency increases hepatic *Lepr* expression in DEN-induced HCC livers**. qPCR analysis of (A) *Il6rα*, (B) *Socs3*, (C) *Timp1*, and (D) *Leprb* (probe spanning exon 18–19 of long *Leprb*) mRNA in whole liver tissue isolated from tumor bearing (DEN-induced HCC) NCD- and HFD-fed IL-6Rα^KO^ and control mice, normalized to Ctrl NCD (n = 3–8, ordinary two-way ANOVA). (E) Serum leptin levels from 8-months-old IL-6Rα^KO^ and control mice in DEN-induced HCC upon NCD and HFD feeding (n = 11–12, ordinary two-way ANOVA). (F) Corresponding body weights of Ctrl and IL-6Rα^KO^ mice at 8 months (n = 11–12, ordinary two-way ANOVA). (G) Schematic representation of mouse mutants used in this study. (H, I) Western blot analyses of pSTAT3 in hepatocytes isolated from indicated mouse mutants stimulated with either (H) 50 ng/ml IL-6 or (I) 1 μg/ml leptin for 15, 30 or 60 min. (J) Time beam of DEN experiments and metabolic phenotyping. HCC = hepatocellular carcinoma, DEN = diethylnitrosamine, NCD = normal chow diet, HFD = high-fat diet. p* ≤ *0*.*05*, p** ≤ *0*.*01*, p*** ≤ *0*.*001*, p**** ≤ *0*.*0001*. Data are means ± Min. and Max.

**Figure 3:**
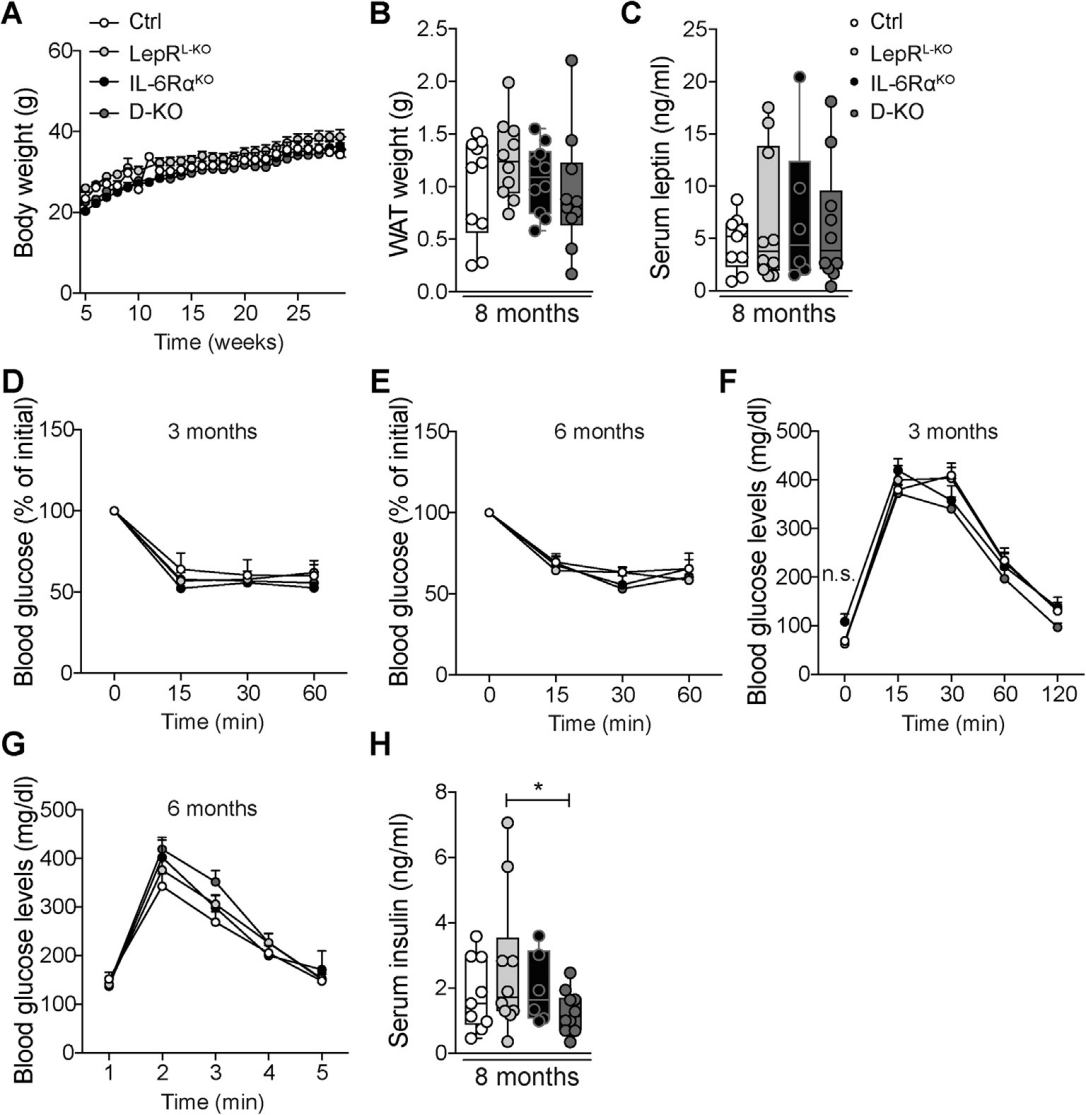
**Hepatic LEPR and whole body IL-6Rα deficiency display only marginal effects on whole body metabolism in DEN-induced HCC**. (A) Body weight curve of Ctrl, LepR^L−KO^, IL-6Rα^KO^, and D-KO (n = 10) mice fed a NCD. (B) Absolute epididymal WAT weight of NCD-fed Ctrl, LepR^L−KO^, IL-6Rα^KO^, and D-KO with 8 months (n = 10, ordinary one-way ANOVA). (C) Serum leptin levels from NCD-fed 6-month-old mice (n = 6–10, ordinary one-way ANOVA). Insulin tolerance determined at (D) 11 weeks of age of Ctrl, LepR^L−KO^, IL-6Rα^KO^, and D-KO (n = 10) and (E) 24 weeks of age of Ctrl (n = 10), LepR^L−KO^ (n = 10), IL-6Rα^KO^ (n = 5), and D-KO (n = 10) (ordinary two-way ANOVA) mice fed a NCD. Values are displayed as % of initial basal blood glucose. Glucose tolerance determined at (F) 12 weeks of age and (G) 25 weeks of age of Ctrl (n = 10), LepR^L−KO^ (n = 10), IL-6Rα^KO^ (n = 5), and D-KO (n = 10) (ordinary two-way ANOVA) mice fed a NCD. (H) Serum insulin levels from NCD-fed 6-months-old mice (n = 6–10, ordinary one-way ANOVA). WAT = white adipose tissue, NCD = normal chow diet. n.s. = not significant. p* ≤ *0*.*05*. Data are means ± Min. and Max (B, C, H) or ± SEM (A, D, E, F, G).

**Figure 4:**
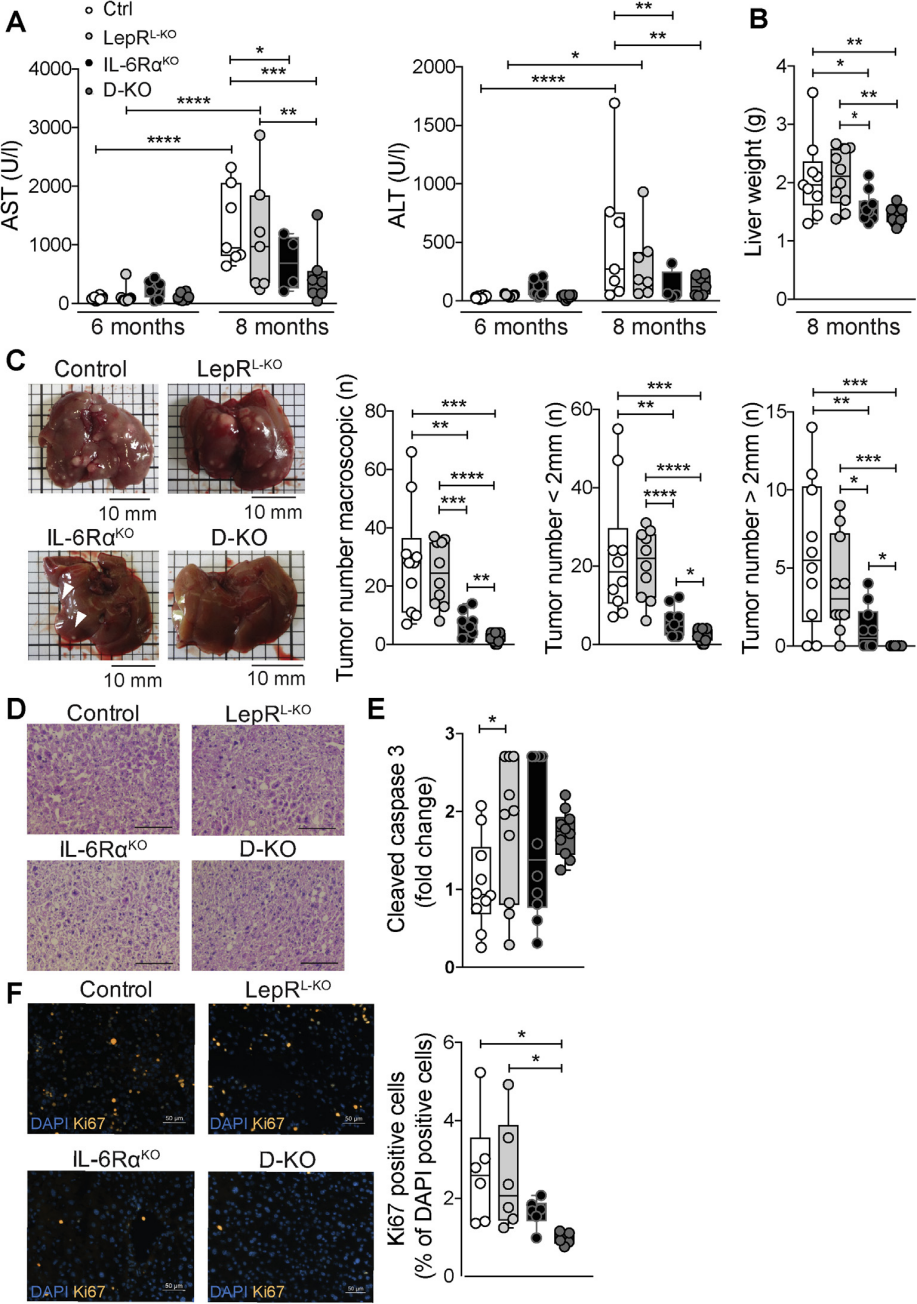
**Hepatic LEPR deficiency further reduces DEN-induced HCC in IL-6Rα**^**KO**^**mice**. (A) Serum AST and ALT levels after 6 and 8 months of age of Ctrl (n = 4–10), LepR^L−KO^ (n = 8–10), IL-6Rα^KO^ (n = 5–9), and D-KO (n = 8–12) mice fed a NCD (ordinary two-way ANOVA). (B) Liver weight after 8 months of age of NCD-fed Ctrl, LepR^L−KO^, IL-6Rα^KO^, and D-KO (n = 9–10, ordinary one-way ANOVA). (C) Tumor burden after 8 months of age of NCD-fed Ctrl, LepR^L−KO^, IL-6Rα^KO^, and D-KO (n = 10, multiple t-test with correction for multiple comparison, Holm-Sidak method). (D) Representative pictures of H&E stainings of non-tumor liver tissue from NCD-fed Ctrl, LepR^L−KO^, IL-6Rα^KO^, and D-KO (n = 3). Scale bar = 100 μm. (E) Fold change of activated caspase 3 in liver lysates isolated from 8-months-old DEN injected NCD-fed mutant mice, normalized to Ctrl (n = 10, ordinary one-way ANOVA). (F) Representative pictures of Ki67 IHC (red) in livers of DEN injected NCD-fed Ctrl, LepR^L−KO^, IL-6Rα^KO^, and D-KO mice and respective quantification (n = 5–6, ordinary one-way ANOVA). Data are presented as percentage of Ki67 positive cells normalized to DAPI (blue) positive nuclei. Scale bar = 50 μm. AST = aspartate aminotransferase, ALT = alanine aminotransferase, NCD = normal chow diet. Data are means ± Min. and Max. p* ≤ *0*.*05*, p** ≤ *0*.*01*, p*** ≤ *0*.*001*, p**** ≤ *0*.*0001*.

Data are presented as means ± Min. and Max. or means ± SEM. Statistical significance was either calculated using a two-tailed unpaired student's T-test (Figure 1A, B), multiple T-tests with correction for multiple comparisons, Holm Sidak method (Figure 4C), ordinary one-way ANOVA with Fisher's LSD test (Figure 3B, C, H, Figure 4B, E, F, Supplementary Fig. 3A, B, C, E, F, Supplementary Fig. 4A, B, C, D, E, F, G, H), or ordinary two-way ANOVA with Fisher's LSD test (Figure 2A, B, C, D, E, F, Figure 3D, E, F, G, Figure 4A, Supplementary Fig. 2A, B). Significance was accepted at the level of p* ≤ *0*.*05*, p** ≤ *0*.*01*, p*** ≤ *0*.*001*, p**** ≤ *0*.*0001*.

## 3 Results

### 3.1 Diet-induced obesity increases basal hepatic STAT3 activation and *Lepr* expression

Diet-induced obesity (DIO) systemically increases the STAT3-activating factors IL-6 and leptin that are both derived from the growing white adipose tissue (WAT). Therefore, IL-6- and leptin-responsive tissues such as liver should increase their basal intracellular signaling. Consistently, basal hepatic STAT3 activation as monitored by phosphorylation at residue Tyr705 was increased in obese mice, although with strong variation (Figure 1A). While the major STAT3 activator in the obese liver is IL-6/IL-6Rα, it could be that leptin also contributes to the increased hepatic STAT3 activation [23]. The effects of leptin on metabolism are predominantly mediated via expression of the LEPR on specific hypothalamic neuronal circuits, but other tissues, such as the liver, also express LEPR [50]. To determine whether hepatic LEPR expression is regulated in obesity, *Lepr* mRNA in the liver was analyzed in mice exposed to NCD or HFD. mRNA expression of the long *Leprb* isoform, which is capable of signaling, was found to be slightly, but significantly increased in whole liver lysates of HFD-fed mice compared to NCD-fed mice (Figure 1B). To further analyze which cell type within the liver increases *Leprb* gene expression, liver sections of NCD- and HFD-fed animals were examined for *Lepr* expression via *in situ* hybridization (ISH) using RNAscope, and macrophages were stained using F4/80 antibody (Figure 1C, Supplementary Fig. 1A). This experiment revealed that besides some F4/80 positive cells, mainly hepatocytes express the *Lepr*. Thus, these experiments suggest that DIO increases *Lepr* mRNA expression in the murine liver, most likely in hepatocytes.

### 3.2 Increased hepatic Lepr expression in IL-6Rα-deficient mice in DEN-induced HCC

We have previously demonstrated that IL-6Rα whole body deficiency protects lean mice from DEN-induced HCC [23]. However, the protection against DEN-induced HCC in IL-6Rα^KO^ mice was not complete and these animals still developed a small number of HCC after DEN injection, although *Il6rα* expression was genetically ablated (Figure 2A) [23]. In light of its essential function in hepatic regeneration and maintenance we assumed that other JAK/STAT3-inducing factors might compensate for IL-6Rα deficiency. In line with this evidence, expression of representative IL-6-mediated STAT3 target genes such as *Socs3* and *Timp1* were largely unaltered in tumor livers of lean IL-6Rα-deficient mice compared to controls (Figure 2B, C). However, HFD feeding increased their expression in control mice when compared to IL-6Rα-deficient mice (Figure 2B, C).

Leptin and IL-6 activate similar downstream signaling pathways and both are increased in circulation upon obesity [60], [61]. Hence, we assessed hepatic *Lepr* mRNA expression as potential compensating factor in IL-6Rα-deficient mice. Interestingly, significantly elevated *Lepr* gene expression was observed in tumor livers of IL-6Rα^KO^ mice under both, lean and obese conditions (Figure 2D). Moreover, despite similar bodyweights, circulating leptin levels were significantly increased in obese IL-6Rα^KO^ mice when compared to obese control mice suggesting that indeed under HFD-feeding leptin might exert compensatory effects in the liver of these mice (Figure 2E, F).

To further decipher the effects of potential compensation of LEPR signaling in IL-6Rα deficiency, we generated experimental double KO (D-KO) mice carrying IL-6Rα inactivation in the whole body and hepatic LEPR deficiency by intercrossing the *Il6rα* complete knock out allele with liver specific ALFP-Cre and *Lepr* floxed alleles (Figure 2G). Of note, we inactivated the *Lepr* hepatocyte-specific instead of a whole body knock out to prevent morbid obesity, which is seen in mice with completely disrupted leptin signaling [47], [49]. As controls LepR^fl/fl^, sensitive to both IL-6 and leptin, LepR^L−KO^ with hepatocyte-specific ablation of the LepR, and whole body IL-6Rα^KO^ mice, which are unable to respond to IL-6, were used. To first investigate the hepatic abilities of the different cohorts to respond to IL-6 and leptin, we isolated hepatocytes and performed *in vitro* stimulation experiments (Figure 2H, I). While we observed phosphorylation of STAT3 in LepR^fl/fl^ and LepR^L−KO^ derived hepatocytes upon IL-6 stimulation for 15, 30, and 60 min, IL-6Rα deficiency completely prevented IL-6-induced STAT3 activation in hepatocytes derived from IL-6Rα^KO^ and D-KO mice as expected (Figure 2H). Interestingly, when stimulating hepatocytes with leptin for 15, 30, and 60 min only IL-6Rα^KO^ hepatocytes were capable to activate STAT3, but not hepatocytes isolated from the other cohorts of mice. Still, the different hepatocytes exhibited the capacity to activate STAT3 upon FCS stimulation as positive control (Figure 2I). Thus, these experiments functionally validate our observation that IL-6Rα^KO^ mice overexpress the LEPR in the liver, thereby underlining our hypothesis that LEPR expression might compensate for IL-6Rα deficiency in HCC development.

To address the effect of additional hepatic LEPR ablation in whole body IL-6Rα-deficient mice on HCC development, we subjected cohorts of Ctrl, LepR^L−KO^, IL-6Rα^KO^ and D-KO mice to the DEN-induced HCC model according to a protocol depicted in Figure 2J, which allows for metabolic as well as oncogenic characterization. DEN *i*.*p*. injection into male mice at postnatal day 15 (P15) leads to hepatic DEN metabolization, thereby damaging hepatocytes, to finally result in compensatory hepatocyte proliferation and HCC development at later stages of life (8 months) with high incidence [62].

### 3.3 Body composition and glucose metabolism remain largely unaltered upon hepatic LEPR inactivation in IL-6Rα-deficient mice

Cohorts of mice were injected at P15 with 25 mg/kg BW DEN and exposed to NCD upon weaning. BW was monitored weekly for 8 months; however, no difference in BW-gain between the genotypes was detected (Figure 3A). In this line, epididymal WAT weight was not altered between the four genotypes at 8 months of age (Figure 3B). Consistently, circulating leptin concentrations were similar in all cohorts of mice (Figure 3C). Thus, these data unequivocally reveal that neither hepatic LEPR deficiency, nor IL-6Rα inactivation, nor ablation of both affect body composition in the DEN model of HCC. Unaltered BW-gain in these cohorts during 8 months of maintenance is unexpected due to a previous publication, which demonstrates that IL-6-deficient mice develop mature onset obesity [63]. However, we never observed alterations in BW-gain between DEN-induced control and IL-6Rα-deficient animals and our data are in line with previous reports that examined IL-6 and IL-6Rα knock out animals in DEN-induced HCC that showed similar BW compared to controls [22], [23]. Otherwise, IL-6Rα has been shown to also bind the closely related IL-6 type cytokine CNTF and this signaling is also abolished in our knock out mice [64].

We further examined insulin sensitivity (Figure 3D and E, Supplementary Fig. 2A and B) and glucose tolerance (Figure 3F and G) in the cohorts of mice at an early (3 months) and a late (6 months) time point of DEN-induced liver cancerogenesis. However, the different genotypes exhibited similar insulin sensitivity and glucose tolerance both early and late in cancerogenesis. Hepatic LEPR and whole body IL-6Rα deficiency did not affect circulating serum insulin levels (Figure 3H). While these experiments are in line with our previous study that verifies unaltered glucose metabolism in whole body IL-6Rα-deficient mice in DEN-induced HCC, mice with hepatocyte-specific IL-6Rα inactivation develop insulin resistance owing to systemic inflammation originating from Kupffer cells [23], [57]. Given the diverse metabolic and inflammatory alterations in mice with conditional IL-6Rα inactivation in hepatocytes, macrophages, T cells, NK cells, and even in the CNS indicates not only individual cell type-specific functions of IL-6 but also an abrogated role of whole body IL-6Rα deficiency on glucose metabolism [57], [65], [66], [67], [68].

Hepatic LEPR, whole body IL-6Rα, and combined deficiencies did not alter body composition, insulin sensitivity, and glucose tolerance in the DEN model of liver cancer *in vivo*. However, leptin and IL-6 signaling might still have an impact on metabolic pathways such as cholesterol homeostasis, triglyceride and fatty acid synthesis, glycolysis/glycogenesis and angiogenesis. We found that the expression of key cholesterol homeostasis genes *Srebp1* and *Srebp2* were decreased in IL-6Rα−deficient mice (single and D-KO) (Supplementary Fig. 3A). In agreement, circulating cholesterol level were reduced in these mice, indicating that IL-6 but not hepatic leptin signaling has an impact on cholesterol homeostasis (Supplementary Fig. 3A). On the one hand, expression of central enzymes of triglyceride synthesis *Dgat1* and *Dgat2* were increased in livers of mice with IL-6Rα deficiency, whereas circulating triglycerides were reduced (Supplementary Fig. 3B). On the other, central lipid metabolism genes such as *Scd1* and *Fasn* were similarly expressed in livers of all genotypes. Only *Pparg* expression was slightly decreased in IL-6Rα^KO^ and D-KO mice when compared to LepR^L−KO^ (Supplementary Fig. 3C). However, this was not significant when compared to Ctrl animals. Ultimately, Oil red O stainings revealed similar little hepatic lipid accumulation in the different mouse mutants (Supplementary Fig. 3D). Thus, our data reveal slightly altered hepatic lipogenesis, in both IL-6Rα^KO^ and D-KO mice, which ultimately fails to affect hepatic lipid content in lean mice.

We have previously shown that IL-6 signaling in hepatocytes regulates glycolysis and glycogenesis via Stat-3-mediated control of glucose 6 phosphatase (G6Pase) and glucokinase (Gck) [57]. Thus, we examined expression of *Gck*, *Pepck*, and *G6Pase* in livers of our mice, revealing that gene expression was largely unaltered (Supplementary Fig. 3E). Of note, in the current study we have used whole body IL-6Rα-deficient mice, whereas in our previous study only hepatocytes were unresponsive to IL-6 in IL-6Rα^L−KO^ mice. There we observed that hepatic IL-6Rα deficiency results in exacerbated systemic inflammation originating from liver-resident Kupffer cells to result in the development of insulin resistance that also affect expression of glycolytic and glycogenic enzymes. Nevertheless, our expression analysis of genes controlling glycolytic flux revealed similar gene expression of *Gck*, *Pepck*, and *G6Pase* in livers of DEN-induced control, LepR^L−KO^, IL-6Rα^KO^ and D-KO mice (Supplementary Fig. 3E).

Leptin and IL-6 have been shown to also regulate angiogenesis [69], [70], [71]. To this end, we have investigated gene expression of *Vegf*, *Egf*, *Egfr*, and *Glut1*, which are essential/associated pro-angiogenic growth factors (Supplementary Fig. 3F) [72], [73]. While this analysis revealed largely unaltered gene expression of angiogenic genes in our mouse cohorts, we concordantly verified this fact via Lectin staining of mouse livers that demonstrated similar stainings of vessels and distribution between the different genotypes (Supplementary Fig. 3G).

Given these results, liver cancer development in such lean cohorts occurs in the absence of large metabolic alterations allowing for the direct comparison between the genotypes. These results are in line with a previous publication that demonstrated largely unaltered metabolic parameters in mice with inactivation of LEPR in all peripheral tissues using tamoxifen inducible Cre recombinase mice [74].

### 3.4 Additional hepatic LEPR deficiency ameliorates HCC tumor burden in the IL-6Rα-deficient background

Given the largely unaltered metabolism between the four cohorts of mice, liver cancer development and progression were analyzed. To monitor DEN-induced HCC progression indirectly via liver damage, we measured aspartate aminotransferase (AST) and alanine aminotransferase (ALT) activities in the serum of 6-month- and 8-month-old mice. While after 6 months of age serum AST and ALT activities were low in all cohorts, indicating no or low HCC, liver damage increased in Ctrl and LepR^L−KO^ mice after 8 months. Interestingly, IL-6Rα^KO^ and D-KO mice displayed significantly reduced AST and ALT levels in the serum (Figure 4A). At 8 months of age, mouse cohorts were sacrificed and analyzed for liver weight and HCC burden. Liver weights were reduced in IL-6Rα^KO^ mice and further decreased in D-KO mice when compared to Ctrl and LepR^L−KO^ mice (Figure 4B). Furthermore, number and size of tumors were determined via macroscopic inspection and distinguished between large (>2 mm) and small (<2 mm) tumors, respectively (Figure 4C). While Ctrl and LepR^L−KO^ mice developed similar HCC burden, IL-6Rα deficiency protected against DEN-induced HCC in both single IL-6Rα^KO^ and D-KO mice (Figure 4C). However, while IL-6Rα^KO^ mice still developed 6.5 tumors on average, additional hepatic LEPR deficiency in D-KO mice further reduced HCC burden to 2 tumors on average. Strikingly, while several IL-6Rα^KO^ mice developed large tumors, such tumors were completely absent in D-KO mice (Figure 4C). However, tissue structure and integrity of non-tumor areas, analyzed by H&E staining, were similar between the genotypes (Figure 4D). In agreement, hepatocyte apoptosis was largely unaltered and variable in livers at this stage, as revealed via an ELISA-based caspase 3 activity assay in liver lysates (Figure 4E). Notably, we found a significant reduction of proliferating hepatocytes (Ki67+) in D-KO animals when compared to Ctrl and LepR^L−KO^ mice. This significant reduction was not detected in single IL-6Rα^KO^ mice (Figure 4F). Since STAT3 downstream signaling induces proliferation, we assumed that STAT3 activation could be further reduced in D-KO mice. However, while *Stat3* mRNA and STAT3 protein levels were similar in all genotypes, its steady state activation in HCC livers as revealed by pSTAT3 western blot and pSTAT3 immunohistochemistry remained largely unaltered even in D-KO mice with high variabilities (Supplementary Fig. 4A, B, C). Given this unexpected finding on unaltered steady state STAT3 activation in HCC livers, it is tempting to speculate that either IL-6/leptin-induced signaling is essential earlier in hepatocarcinogenesis such as acutely after DEN injection or that other signaling pathways are affected in D-KO mice. In line with this evidence, IL-6 and leptin also signal via PI3K/Akt to the mammalian target of rapamycin (mTOR), which, in turn, regulates protein synthesis via eukaryotic translation initiation factor 4E (eIF4E) [75], [76]. eIF4E was also shown to induce proliferation and promote tumorigenesis [77]. Thus, we performed western blots and qPCRs to examine whether eIF4E is differentially activated in our mouse cohorts. Accordingly, western blot analysis using p-eIF4E, eIF4E and β-actin antibodies revealed a significant reduced activation of eIF4E in IL-6Rα^KO^ and D-KO HCC livers (Supplementary Fig. 4D). Activation of eIF4E in tumors regulates not only proliferation, but also evasion from apoptosis, fibrosis and metastasis and targeting of the eIF4F complex is of particular interest in developing new cancer treatment strategies [78], [79]. While expression for genes regulating proliferation and apoptosis were not changed between the genotypes, genes upregulated in fibrosis and metastasis were reduced in IL-6Rα^KO^ and D-KO mice (Supplementary Fig. 4E, F, G, H). However, the early termination of our HCC experiments at 8 months does not allow for further investigating alterations in fibrosis and metastasis.

Collectively, our experiments show that hepatic LEPR exerts a compensatory function in IL-6Rα-deficient mice in DEN-induced liver cancer development largely independent of changes in whole body metabolism. These results may pave the way for potential therapeutic approaches targeting the liver/HCC to simultaneously inhibit hepatic leptin and IL-6 signaling or even downstream signal transduction.

## 4 Discussion

Leptin- and IL-6-induced signaling via their respective receptors plays a major role not only in the control of energy homeostasis, but also under pathological conditions such as liver cancer beyond dispute. However, the diverge expression patterns of receptors, their varying cell type-specific functions, as well as different signaling capabilities render experimental setups difficult to study individual, compensating, and synergistic actions of these factors in obesity-associated disorders. For instance, In DIO, mice develop neuronal leptin resistance characterized by the inability of leptin to reduce food intake while central IL-6 sensitivity is maintained to reduce food consumption in obese mice via a sIL-6R trans signaling mechanism [68], [80]. Furthermore, the different outcomes of complete genetic disruptions of these signaling pathways prevent, or at least complicate the direct comparison of such mouse mutants in disease models. Leptin- (*ob/ob*) or LEPR-deficient (*db/db*) mice for instance are morbidly obese due to uncontrolled food intake, whereas animals deficient for IL-6 develop mature onset obesity [47], [48], [49], [63]. Thus, carefully and properly controlled investigations, as well as genetic assessments of cell type-specific functions in basic research will set the ground to decipher the different roles of these cytokines in disease states.

Hence, we examined the role of leptin and IL-6 signaling in HCC development, using mice with hepatic LEPR ablation and whole body IL-6Rα deficiency, which did not affect body composition and glucose homeostasis compared to control mice in the DEN-model. This further allows for accurate assessment of liver cancerogenesis without unpredictable side effects, such as morbid obesity and disturbed eating behavior. In fact, the unaltered BW-gain, insulin sensitivity, and glucose tolerance, covering the complete experimental time frame, suggest a minor role of hepatic LEPR and IL-6-induced processes on metabolism under lean conditions. We have identified IL-6/leptin evoked signaling as an effector of lipogenesis that did not affect hepatic lipid accumulation in our model using lean animals. Nevertheless, this might be different under obese conditions.

However, our finding of increased hepatic *Lepr* expression in obese and IL-6Rα-deficient animals suggests functions for leptin signaling in the liver independent of its role in controlling whole body metabolism. While the role of IL-6 in promoting function in liver cancer is well documented, less is known about leptin signaling in HCC development. A previous report demonstrated an increase of *Lepr* mRNA expression in the brain of diet-induced obese mice [81]. In contrast, however, *Lepr* gene expression levels were reduced in hypothalamus and liver of rats exposed to a short term DIO protocol [82]. DIO causes leptin resistance [83], and the increased LEPR expression could be derived by compensatory mechanisms in order to overcome the blunting of intracellular leptin signaling. Hepatic LEPR expression increases upon leptin injection and food deprivation [50]. Notably, we have shown previously in mice with inducible insulin receptor ablation that the genetically caused insulin resistance drastically increased hepatic LEPR expression that at least in part absorbed the lack of hepatic insulin action in these mice [84]. Furthermore, mice with genetic insulin resistance in liver increase hepatic expression of LEPR and demonstrate an 80-fold increase in circulating forms of LEPR [85]. Other studies have described shedding and alternative splicing of a soluble form of LEPR derived from the liver [50] that modulates circulating leptin levels and possibly its biological activity [86]. However, examining serum of obese humans revealed decreases in soluble LEPR isoforms [87], [88]. Therefore, our results showing significantly increased hepatic RNA of the long form of *Lepr*, capable of transmitting leptin's action into cells of obese and IL-6Rα-deficient mice, suggest a hepatocyte-intrinsic cell-autonomous mechanism rather than a systemic shift in leptin action. Interestingly, only IL-6Rα-deficient hepatocytes, but not Ctrl, Lepr-deficient, and double-deficient hepatocytes reacted to *in vitro* leptin stimulation indicating that genetic IL-6Rα disruption sensitizes hepatocytes to other STAT3-inducing factors as a presumably compensating mechanism. This is in line with our previous publication in which we postulated a STAT3-inducing factor in obesity to adopt IL-6 signaling in HCC development of IL-6Rα-deficient mice [23]. In this study, we have already revealed that IL-6Rα deficiency in lean mice was able to reduce DEN-induced HCC burden, but failed to completely prevent liver cancer development [23]. In detail, the IL-6Rα^KO^ mice in the present and in the previous study developed between 5 and 10 tumors whereby up to 3 were considered to be larger than 2 mm in diameter. In the present study, we have revealed that additional hepatic LEPR deficiency in the liver further reduces HCC burden in lean IL-6Rα-deficient animals whereas mice with single hepatic LEPR deficiency showed only marginal effects on HCC development. Strikingly, the D-KO mice displayed a full protection against the development of large tumors and a reduced total tumor burden compared to IL-6Rα single knockouts, which grants LEPR signaling a compensating tumor-promoting effect in DEN induced HCC development. This finding is further supported by the fact that leptin can induce cancer cell survival, proliferation, invasion, and migration, as well as tumor angiogenesis to promote progression of breast, endometrial and pancreatic cancer [51]. The assurance of nutrient supply for solid tumors is critically dependent on angiogenesis [89] and both leptin and IL-6 have been reported as pro-angiogenic factors [90]. However, we did not identify major alterations in angiogenesis in our hepatic LEPR-, IL-6Rα-, or double-deficient mice. Interestingly, although we revealed decreased Ki67 positive/proliferating cells in D-KO mice, this was surprisingly not accompanied by decreased steady state pSTAT3 levels. While this finding needs further evaluation, it might be that IL-6/leptin-induced STAT3 is required earlier in DEN-induced HCC e.g. in tumor initiation or during progression. Conversely, other STAT3-inducing factors or other downstream signaling events such as PI3K/Akt/mTOR, that are not well investigated yet, might impact HCC. In line with this evidence, eIF4E, a molecule that is part of the eIF4F complex, which mediates translational control and is strongly associated with tumorigenesis, is also affected by HFD-induced obesity thereby supporting the link between obesity and HCC [79], [91], [92]. Leptin as well as IL-6 can activate mTOR through downstream PI3K/Akt signaling [93], [94], and mTOR indirectly activates eIF4E, which, in turn, stimulates translation of mRNAs encoding for proliferation and anti-apoptotic factors [95]. Therefore, a reduced IL-6/leptin-evoked signaling capacity via Akt/mTOR/eIF4E axis could contribute to reduced HCC burden in hepatic LEPR and IL-6Rα double-deficient mice. Given that HFD-induced obesity and IL-6Rα deficiency both increase hepatic Lepr expression suggests that obese animals would profit even more from genetic IL-6Rα and hepatic Lepr deficiency in the DEN-induced HCC protocol. However, the mouse cohort characterized in this study prohibits such experiments since the mixed C57/BL6X129 background provides resistance to diet-induced obesity. In particular, we assured similar genetic backgrounds in our lean mouse cohorts as the parents descended from the same intercross. Nevertheless, the overlapping downstream signaling of leptin and IL-6 (such as Stat3 and mTOR/eIF4E), as well as our findings that hepatic leptin signaling can partially compensate for IL-6Rα deficiency in HCC development, prompts further investigation of intracellular signaling cascades the therapeutic inhibition of which might help to prevent cancer deaths of millions of affected people per year. However, the therapeutic inhibition of IL-6- and leptin-evoked signaling via Stat3 inhibitors or rapamycin in cancer therapy might still be a challenge due to systemic effects. Thus, basic research together with commercial institutions must find a way to specifically target such signaling pathways in cancer cells. Promising therapeutic approaches such as blockade of IL-6 trans signaling via sGP130Fc may have the potential for combinatory therapies with similar pharmacological perspectives against the leptin receptor to ultimately reduce deaths from HCC.
