# Supplementary material for: Hepatic leptin receptor expression can partially compensate for IL-6Rα deficiency in DEN-induced hepatocellular carcinoma
Source: Mol Metab. 2018 Sep 5;17:122–33. doi: 10.1016/j.molmet.2018.08.010 (PMC6197506; doi:10.1016/j.molmet.2018.08.010)
Supplement: Multimedia component 1 [file mmc1.pdf]

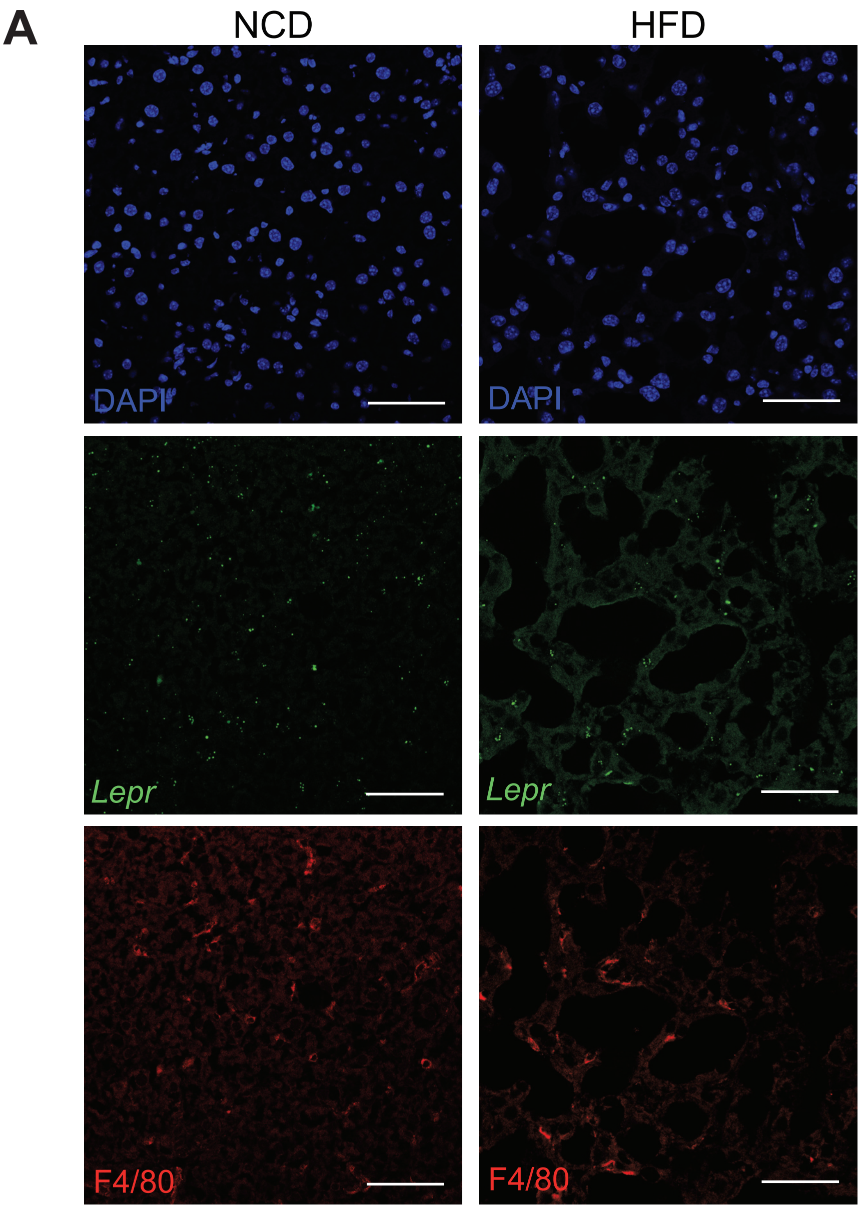

Supplementary Figure 1

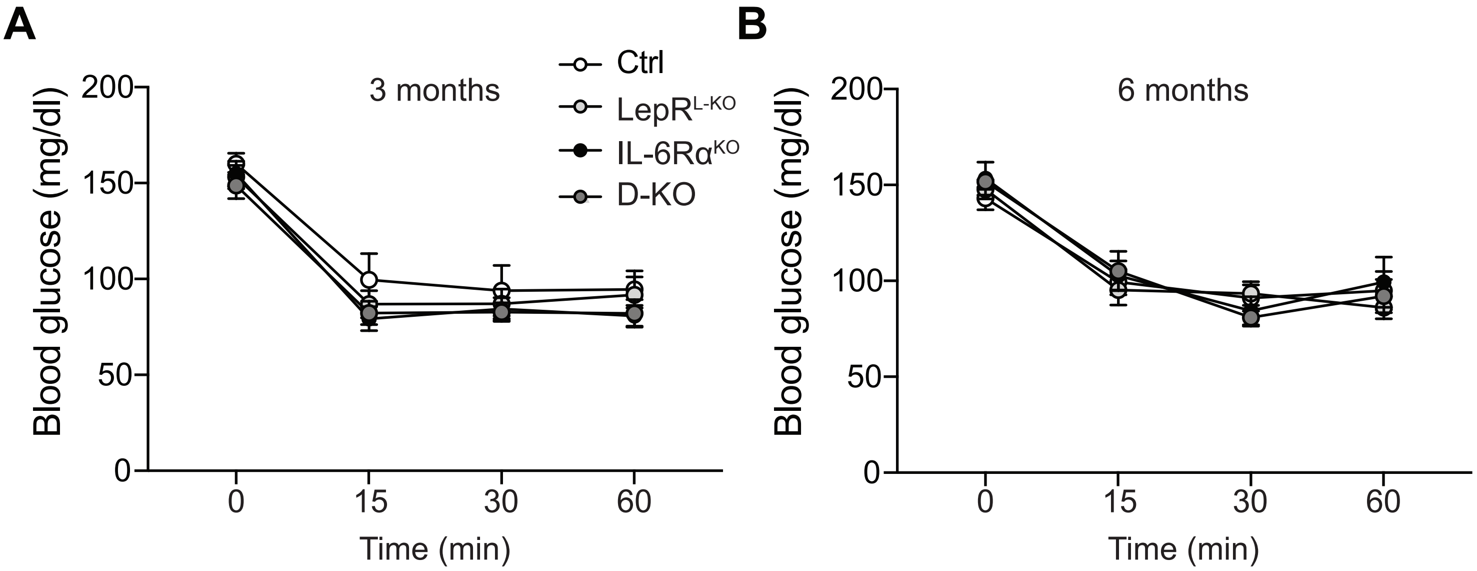

Supplementary Figure 2

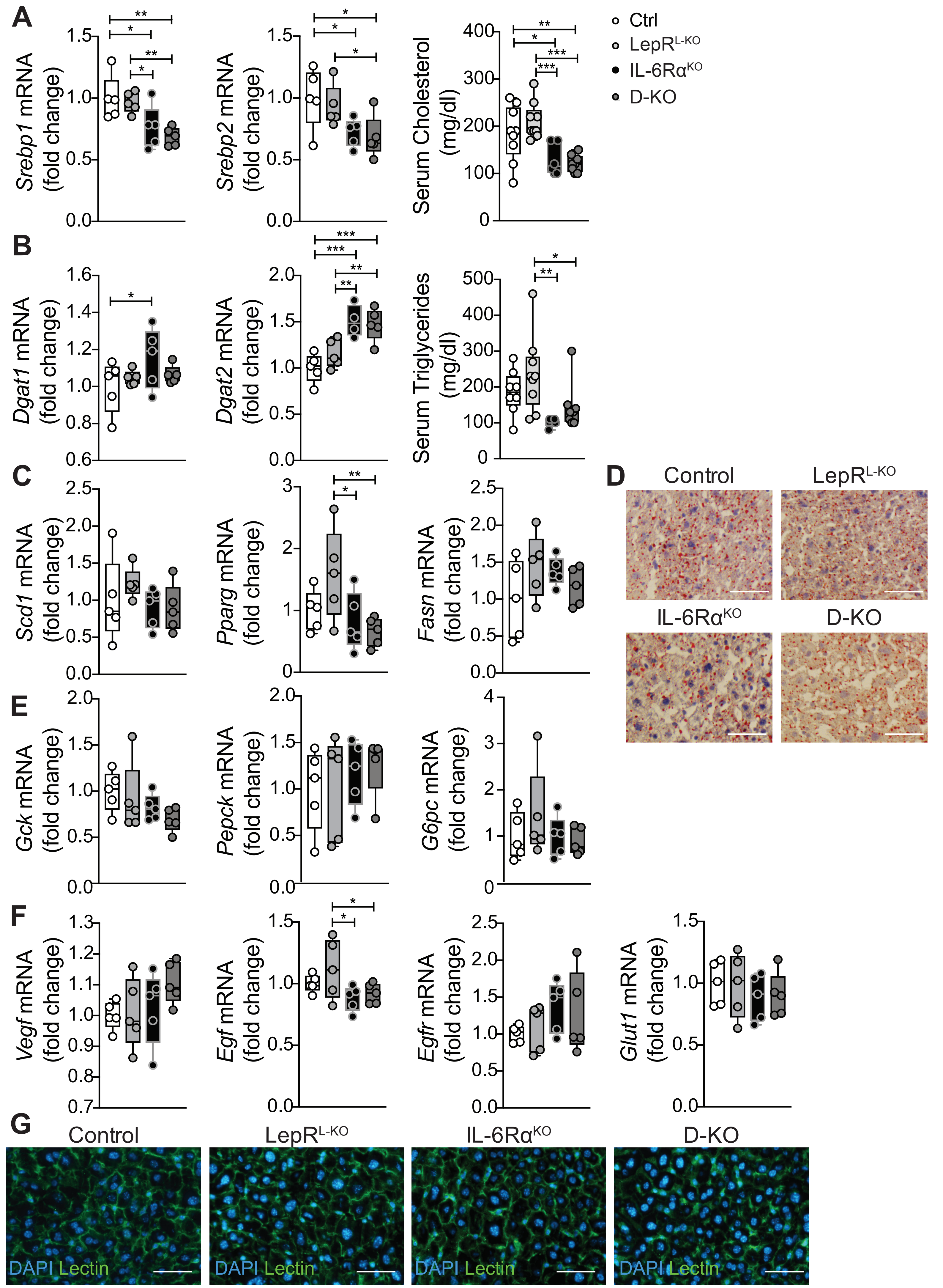

Supplementary Figure 3

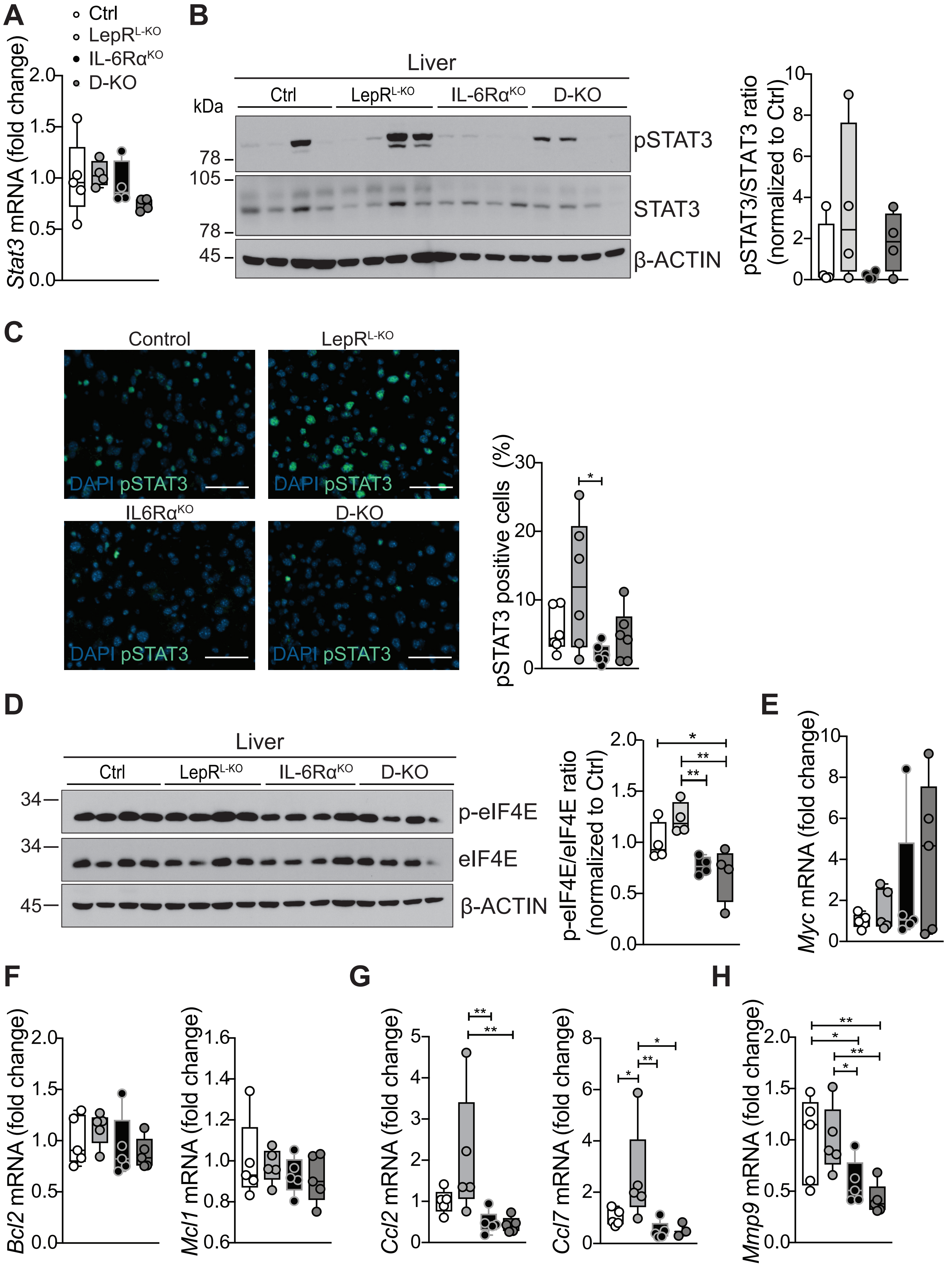

Supplementary Figure 4

Supplementary Figure 1: Hepatocytes and F4/80 positive cells express *Lepr* mRNA under both, NCD and HFD conditions. (A) *Lepr* RNAscope in situ hybridization (ISH) (green) and F4/80 immunohistochemistry (IHC) (red). Different single channel images derived from Fig. 1 C. Nuclei were labeled with DAPI (blue). Scale bar = 50 µm.

Supplementary Figure 2: Unaltered insulin tolerance upon whole body IL-6Rα, hepatic LepR, or double deficiency. Insulin tolerance determined at (A) 11 weeks of age and (B) 24 weeks of age of Ctrl (n = 10), LepR^{L-KO} (n = 10), IL-6Rα^{KO} (n = 5), and D-KO (n = 10) (ordinary two-way ANOVA) mice fed a NCD. Values are measured blood glucose levels in mg/dl 0, 15, 30 or 60 minutes after insulin injection and provide the basis for Fig. 3 D, E.

Supplementary Figure 3: Minor role of hepatic LEPR and IL-6Rα in metabolic flux and angiogenesis. (A) qPCR analysis of key cholesterol homeostasis genes *Srebp1* and *Srebp2* and cholesterol levels in the serum of 8-month-old Ctrl, LepR^{L-KO}, IL-6Rα^{KO}, and D-KO mice (qPCR n = 5, normalized to Ctrl; Serum analysis n = 6-9, ordinary one-way ANOVA). (B) qPCR analysis of central triglyceride synthesis genes *Dgat1* and *Dgat2* and triglyceride levels in the serum of 8-month-old Ctrl, LepR^{L-KO}, IL-6Rα^{KO}, and D-KO mice (qPCR n = 5, normalized to Ctrl; Serum analysis n = 6-9, ordinary one-way ANOVA). (C) qPCR analysis of genes involved in lipid metabolism *Scd1*, *Pparg*, and *Fasn2* of 8-month-old Ctrl, LepR^{L-KO}, IL-6Rα^{KO}, and D-KO mice, normalized to Ctrl (n = 5, ordinary one-way ANOVA). (D) Representative pictures of liver Oil red O stainings from Ctrl, LepR^{L-KO}, IL-6Rα^{KO}, and D-KO mice (n = 3). Scale bar = 50 µm. (E) qPCR analysis of key glycolysis/glycogenesis genes *Gck*, *Pepck*, and *G6pc* of 8-month-old Ctrl, LepR^{L-KO}, IL-6Rα^{KO}, and D-KO mice, normalized to Ctrl (n = 5, ordinary one-way ANOVA). (F) qPCR analysis of pro-angiogenic genes *Vegf*, *Egf*, *Egfr* and *Glut1* of 8-month-old Ctrl, LepR^{L-KO}, IL-6Rα^{KO}, and D-KO mice, normalized to Ctrl (n = 5, ordinary one-way ANOVA). (G) Representative pictures of liver Lectin stainings from Ctrl, LepR^{L-KO}, IL-6Rα^{KO}, and D-KO mice (n = 3). Scale bar = 50 µm. Data are means ± Min. and Max. $p^* \leq 0.05$, $p^{**} \leq 0.01$, $p^{***} \leq 0.001$, $p^{****} \leq 0.0001$.

Supplementary Figure 4: Hepatic LEPR- and whole body IL-6Rα-deficiency does not alter STAT3 activation but reduces eIF4E phosphorylation. (A) qPCR analysis of *Stat3* in the liver of 8-month-old Ctrl, $LepR^{L\text{-}KO}$, $IL\text{-}6R\alpha^{KO}$, and D-KO mice, normalized to Ctrl (n = 4-5, ordinary one-way ANOVA). (B) Western blot analysis of basal pSTAT3 levels in whole liver lysates isolated from 8-month-old Ctrl, $LepR^{L\text{-}KO}$, $IL\text{-}6R\alpha^{KO}$, and D-KO mice (n = 4) and respective western blot quantification (ordinary one-way ANOVA). pSTAT3/STAT3 ratio normalized to Ctrl expression. (C) Representative pictures of pSTAT3 IHC (green) in livers of DEN injected NCD-fed Ctrl, $LepR^{L\text{-}KO}$, $IL\text{-}6R\alpha^{KO}$, and D-KO mice and respective quantification (n = 5-6, ordinary one-way ANOVA). Data are presented as percentage of pSTAT3 positive cells normalized to DAPI (blue) positive nuclei. Scale bar = 50 µm. (D) Western blot analysis of basal p-eIF4E levels in whole liver lysates isolated from 8-month-old Ctrl, $LepR^{L\text{-}KO}$, $IL\text{-}6R\alpha^{KO}$, and D-KO mice (n = 4) and respective western blot quantification (ordinary one-way ANOVA). p-eIF4E/eIF4E ratio normalized to Ctrl expression. (E-H) qPCR analysis of (E) *Myc*, (F) *Bcl2* and *Mcl1*, (G) *Ccl2* and *Ccl7*, (H) *Mmp9* in the liver of 8-month-old Ctrl, $LepR^{L\text{-}KO}$, $IL\text{-}6R\alpha^{KO}$, and D-KO mice, normalized to Ctrl (n = 4-5, ordinary one-way ANOVA). Data are means ± Min. and Max. $p^* \leq 0.05$, $p^{**} \leq 0.01$.
